# Cardiopulmonary resuscitation during hyperbaric oxygen therapy: a comprehensive review and recommendations for practice

**DOI:** 10.1186/s13049-023-01103-y

**Published:** 2023-10-23

**Authors:** Jan Schmitz, Felix Liebold, Jochen Hinkelbein, Sophia Nöhl, Serge C Thal, Timur Sellmann

**Affiliations:** 1 Department of Anesthesiology and Intensive Care Medicine, Faculty of Medicine, University Hospital of Cologne, University of Cologne, 50937 Cologne, Germany; 2 Department of Sleep and Human Factors Research, Institute of Aerospace Medicine, German Aerospace Center, 51147 Cologne, Germany; 3 German Society of Aerospace Medicine, 80331 Munich, Germany; 4 Department of Anesthesiology and Intensive Care Medicine, University Hospital of Leipzig, 04103 Leipzig, Germany; 5 University Department of Anesthesiology, Intensive Care Medicine and Emergency Medicine, Johannes Wesling Klinikum Minden, Ruhr-University Bochum, 32429 Minden, Germany; 6 Department of Anesthesiology I, University Witten/Herdecke, 58455 Witten, Germany; 7 Department of Anesthesiology and Intensive Care Medicine, University Hospital Wuppertal, University Witten/Herdecke, 42283 Wuppertal, Germany; 8 Department of Anesthesiology and Intensive Care Medicine, Ev. Bethesda Hospital Duisburg, 47053 Duisburg, Germany

**Keywords:** Decompression Chamber, CPR, Airway Management, Critical care, Hyperbaric oxygen therapy (HBOT)

## Abstract

**Background:**

Cardiopulmonary resuscitation (CPR) during hyperbaric oxygen therapy (HBOT) presents unique challenges due to limited access to patients in cardiac arrest (CA) and the distinct physiological conditions present during hyperbaric therapy. Despite these challenges, guidelines specifically addressing CPR during HBOT are lacking. This review aims to consolidate the available evidence and offer recommendations for clinical practice in this context.

**Materials and methods:**

A comprehensive literature search was conducted in PubMed, EMBASE, Cochrane Library, and CINAHL using the search string: “(pressure chamber OR decompression OR hyperbaric) AND (cardiac arrest OR cardiopulmonary resuscitation OR advanced life support OR ALS OR life support OR chest compression OR ventricular fibrillation OR heart arrest OR heart massage OR resuscitation)”. Additionally, relevant publications and book chapters not identified through this search were included.

**Results:**

The search yielded 10,223 publications, with 41 deemed relevant to the topic. Among these, 18 articles (primarily case reports) described CPR or defibrillation in 22 patients undergoing HBOT. The remaining 23 articles provided information or recommendations pertaining to CPR during HBOT. Given the unique physiological factors during HBOT, the limitations of current resuscitation guidelines are discussed.

**Conclusions:**

CPR in the context of HBOT is a rare, yet critical event requiring special considerations. Existing guidelines should be adapted to address these unique circumstances and integrated into regular training for HBOT practitioners. This review serves as a valuable contribution to the literature on “CPR under special circumstances”.

## Introduction

Hyperbaric oxygen therapy (HBOT) is a therapeutic modality involving the inhalation of near 100% oxygen at pressures exceeding sea level atmospheric pressure (> 100 kilopascals (kPa)) [1]. HBOT has emerged as the standard of care for numerous acute and chronic medical conditions including decompression sickness, carbon monoxide poisoning, chronic wound care, delayed radiation injury, and other conditions necessitating hyperoxygenation [2][3]. The therapy addresses decompression sickness and gas embolism by increasing pressure, reducing the gas bubble size, and subsequently enhancing tissue oxygenation [4]. This results in elevated oxygen levels in the blood (hyperoxemia) and tissues (hyperoxia) [5]. Table 1 lists additional physiological aspects of HBOT.

In recent years, the application of HBOT has expanded to treat critically and chronically ill patients, presenting new challenges and potential benefit [2]. Given the critical nature of patients requiring HBOT, and the numerous potential side effects associated with the therapy, adverse events are relatively common, with the possibility of rapid patient deterioration [2]. Medical emergencies, including cardiac arrest (CA), may arise during HBOT, necessitating structured management and mental preparation that considers the unique aspects of the hyperbaric environment, including safety aspects [6] [7].

The risk of CA during HBOT affects both acutely and chronically ill patients, with decompensation possible due to the altered atmospheric conditions. Consequently, the structural and environmental differences in a pressure chamber must be acknowledged [6]. HBOT is typically administered in either a monoplace or a multiplace chamber, with the former involving compression and the latter requiring patients to breathe oxygen through a face mask or an endotracheal tube [8]. Depending on the specific indication and corresponding protocol, HBOT sessions can range from several minutes to hours, with treatment frequencies varying from one to three times daily [9][6].

In accordance with the individual treatment protocols, patients inhale 100% oxygen to maximize therapeutic efficacy while also incorporating periods of 21% oxygen to mitigate the risk of oxygen toxicity. Elevated oxygen levels inside of the chamber pose a fire hazard, which has been documented in previous studies [10] [11]. Consequently, the safety of defibrillation during HBOT has been a topic of extensive debate and may impact CPR outcomes in this context [2]. Breathing high-pressure oxygen can lead to oxygen toxicity [11], potentially harming patients and complicating CA treatment. Despite the absence of concrete data on CPR incidence during HBOT, only a limited number of case reports has been published to date.

This narrative review delves into specific aspects of CPR in the context of HBOT. While CPR associated with HBOT appears to be relatively uncommon, this review consolidates recommendations and lessons gleaned from individual case reports, focusing on essential components of CA treatment during HBOT.

**Table 1:** Physiological aspects of hyperbaric oxygen therapy (HBOT)

**Definition** [12]		Treatment of a disease or medical condition by the inhalation of near-100% medical grade oxygen at pressures greater than 1 ATA			
**Physiology** [6, 12, 13]		To enrich plasma with oxygen, maximizing tissue oxygenation. As the oxygen is in solution, it can reach physically obstructed areas and can also enable tissue oxygenation even with impaired haemoglobin oxygen carriage (such as in carbon monoxide poisoning and severe anaemia).			
**Indications** [7, 12, 13]		♣ Air or gas embolism ♣ Carbon monoxide poisoning; cyanide poisoning; smoke inhalation ♣ Clostridial myostitis and myonecrosis (gas gangrene) ♣ Compartment syndromes and other acute traumatic peripheral ischaemia ♣ Decompression sickness ♣ Exceptional blood loss anaemia ♣ Intracranial abscess ♣ Refractory osteomyelitis ♣ Delayed radiation injury (soft tissue and bony necrosis) ♣ Thermal burns			
**Physiological pressure/oxygen correlation according to Sahni et al. **[14]					
**Total pressure**		**Content of oxygen dissolved in** **blood plasma (vol%)**			
*ATA*	*mmHg*	*Breathing Air*	*100% Oxygen*		
1	760	0.32	2.09		normal atmospheric pressure
2	1520	0.81	4.44		pressure during HBOT
2.5	1900	1.06	5.62		
3	2280	1.31	6.8		
> 3	only applied when oxygen fraction is reduced because of risk of oxygen toxicity				
**Adapted treatment protocol (according to U.S. Navy Treatment **[15])				Monoplace (single person): Compression rate 18 kPa/min to 180 kPa, air filled (21%) chamber for approximately 105 min. Afterwards decompression rate 9 kPa/min to 90 kPa for 170 min in oxygen filled (100%) chamber. Decompression rate 9 kPa/min to ambient pressure. Total treatment time: 275 min.	
				Multiplace: Compression rate 18 kPa/min to 180 kPa for 75 min. Afterwards decompression rate 3 kPa/min to 90 kPa for 150 min. Decompression rate 9 kPa/min to ambient pressure. Oxygen breathing is interspersed (every 30 min) with short periods of air breathing (5 min) to lower risk of oxygen toxicity. Total treatment time: 295 min.	
**Decompression rate in case of CPR**				According to possibility to perform high-quality CPR: If possible, consider protocol-based decompression. If CPR is not possible, consider maximum decompression rate (technically according to type of pressure chamber). Recommendation of UHMS (12): Ability to decompress a Class B monoplace chamber from 3 ATA to surface in less than 2 min. Start CPR as soon as possible and safe.	

ATA = atmospheres absolute; CPR = cardiopulmonary resuscitation; HBOT = hyperbaric oxygen therapy; kPa/min = kilopascal per minute; mmHg = millimetres of mercury; vol% = volume percent; UHMS = Undersea and Hyperbaric Medical Society

## Materials and methods

### Search strategy

We performed a comprehensive literature search in PubMed, EMBASE, Cochrane Library and CINAHL up to January 2023 to gather recent data on cardiac arrest in hyperbaric environments. A search string was utilized (**see** appendix) with PubMed searches conducted within “All Fields”, in EMBASE within “Broad search”. EMBASE subsequently refined our initial search string specifically (**see** appendix). Cochrane Library was searched within “Title Abstract Keyword”, and word variations were also explored. In CINAHL, we employed the “Boolean/Phrase” search mode and expanded our search by using additional filters, such as “Also search within the full text of articles” and “Apply equivalent subjects”. When possible, we applied a language filter to display articles exclusively in English and German. In addition to the primary search using the above-mentioned strategy, we partially screened articles identified as similar. Articles discovered randomly during our general search on the topic, along with book chapters and secondary literature, were also included. This study adhered to the Declaration of Helsinki and received approval from the Ethics Committee of the University of Witten/Herdecke (Nr. S-261/2022). As this work was designed as a narrative review, no database registration was performed.

### Inclusion criteria

The primary inclusion criteria encompassed articles describing cardiac arrest, cardiopulmonary resuscitation or defibrillation during HBOT in a decompression chamber. Both patient cases and general information on CPR in the hyperbaric environment were considered. We also included articles containing any other supplementary relevant to our topic. All types of articles were eligible for inclusion, irrespective of their publication year.

### Exclusion criteria

Articles that did not align with the content of our inclusion criteria were excluded. Additionally, we excluded full-text articles written in languages other than English or German.

### Screening procedure

Following initial record identification, a preliminary selection was conducted by screening article titles and search terms highlighted in abstracts. Duplicates were removed after preselecting all articles. The preselected reports were then tabulated and independently assessed for relevance by two authors using color codes (red - irrelevant, yellow - possibly relevant, green - definitely relevant). Article deemed irrelevant by both authors were excluded. In case of different ratings, a third author served as a referee and provided an additionally rating. All reports classified as possibly or definitely relevant were sought for retrieval.

Full-text articles were independently screened by two authors, focusing on study type, indication for HBOT, hyperbaric chamber type, onset of CPR, data on chest compression, airway management, defibrillation, medication and other pertinent findings related to the hyperbaric environment. After screening, full-texts were color-coded based on their relevance, determining which articles were included.

Upon final inclusion decision, two tables summarizing the most important contents were created. Table 2 encompasses all articles describing reported cases of CPR in the pressure chamber, while Table 3 presents papers with additional data on CPR that do not report factual resuscitation in the hyperbaric environment but are deemed useful for the research question. These tables are provided in the results section.

## Results

Our search strategy identified a total of 10.223 publications (PubMed: n = 3739, EMBASE: n = 5403, Cochrane Library: n = 558, CINAHL: n = 523). After the initial screening, we excluded 10.126 records due to lack of relevant content to our research topic. We sought retrieval for the remaining 97 reports, of which we removed 39 duplicates. One report could not be retrieved, leaving 57 full articles identified through our search string for eligibility assessment. In addition to the search string results, we included 25 supplementary articles and book chapters as of January 10th, 2023.

**Fig. 1:**
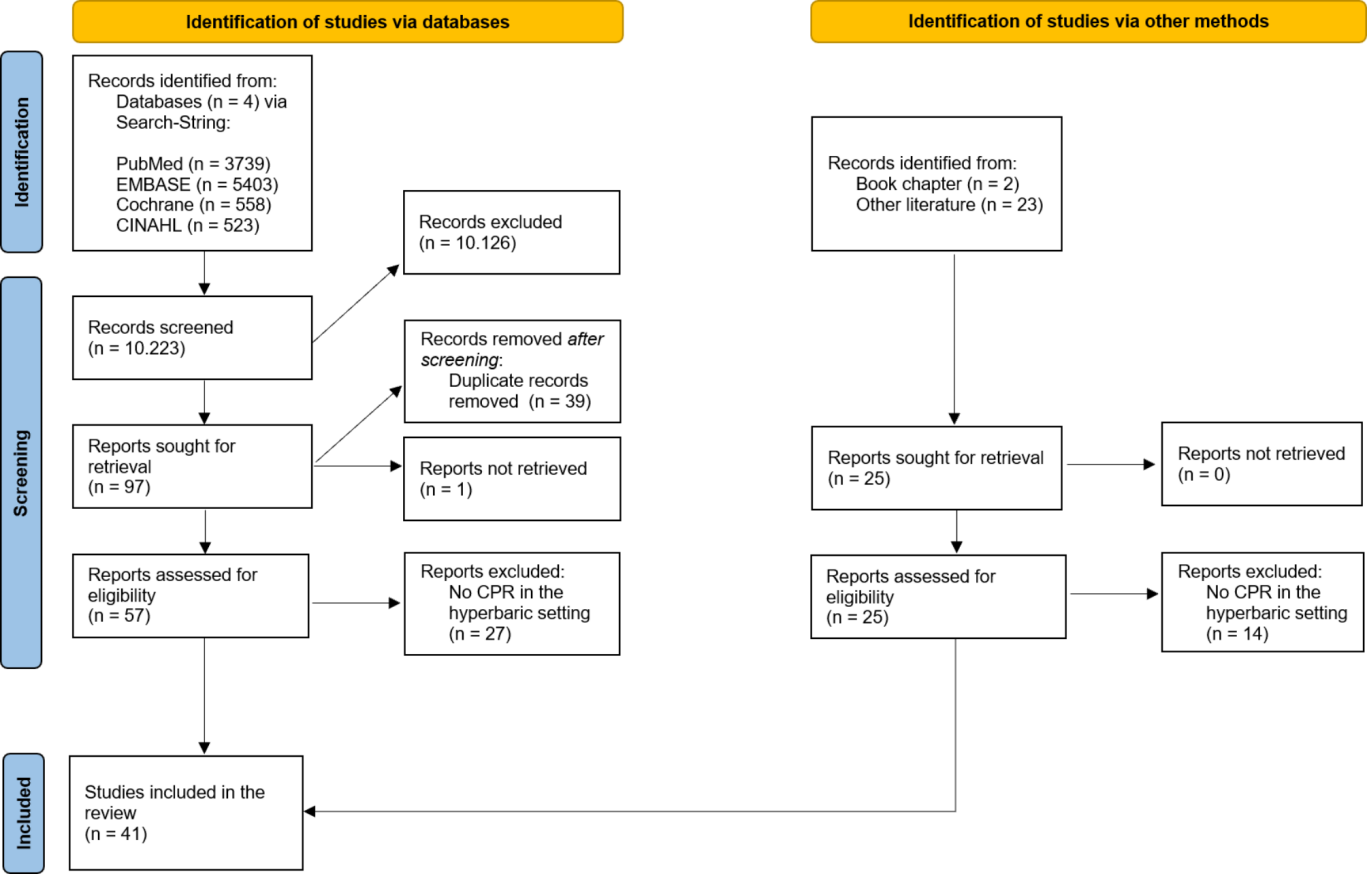
PRISMA (Preferred Reporting Items for Systematic Reviews and Meta-Analyses) Flowchart (according to Page et al. [46])

Upon screening the 82 full texts by two authors, 41 reports were excluded as they did not describe cardiac arrest, CPR, or defibrillation during HBOT in a hyperbaric environment. Consequently, we included 41 articles in our review, all of which were attributable to PubMed and EMBASE. No primary search results were derived from CINAHL or the Cochrane Library, although some duplicate articles were found in CINAHL. Figure 1 presents a PRISMA (Preferred Reporting Items for Systematic Reviews and Meta-Analyses) flow chart of the search strategy.

A total of 18 articles, primarily case reports, described actual CPR events in a hyperbaric pressure chamber, with CPR or defibrillation performed on 22 patients during HBOT. These data are summarized in Table 2. Nine of these cases took place in multiplace hyperbaric chambers, and one case each in a monoplace chamber and a deck decompression chamber. In the other eleven cases the type of pressure chamber was not specified.

**Table 2:** Reported cases of CPR during hyperbaric oxygen therapy (HBOT)

Author, Year, Country, Location	Cases (n)	Age (years)	Sex (m/f)	Indication for HBOT and cause of CPR	Iatrogenic (Y/N)	CPR during HBOT	Outcome
**Bayrakci, 2008** [47] Turkey, Ankara	1	7	m	-CO-intoxication due to CO seepage	N	-90 min of 2nd HBOT on 2nd day after accident	-brain dead, organs were donored
**Burcharth, 2012** [48] Denmark, Copenhagen	1	50	f	-laparoscopic cholecystectomy + liver cyst fenestration -VAE, suspected AAE	Y	-CA at 243 min	-discharge w/o complications POD day 14
**Dieterich, 2016** [6] Germany, Murnau	1	89	f	-gas gangrene after dynamic hip screw -septic shock	Y	-During HBOT	-fatal
**Erdmann, 2009** [49] Germany, Murnau	1	22	m	-CO-intoxication (suicidal) -hypothermia, lactic acidosis, hyperkalemia	N	-transition into 2nd decompression	-no neurological sequelae 3 months after treatment
**Frye, 2007 **[50] USA, Houston (Texas)	1	13	m	-pulmonary valve replacement -AGE	Y	-during HBOT	-no brainstem reflexes, no spontaneous breathing
**Kjeld, 2012 ** [20] Denmark, Copenhagen	1	52	f	-laparoscopy, CCE liver cyst marsupialization -postoperative PEA due to VGE, later VF with CA	Y	-during decompression (pressure lowered to 1.9 ATA after 2,5 h)	-full recovery POD 4
**Lalieu, 2020** [51] Netherlands, Raamsdonksveer	1	37	m	-non-healing venous leg ulcer -acute COT with seizure tachy-/ apnea	N	-last minute of decompression	-fatal
**Machicado, 2013** [52] USA, Houston (Texas)	1	69	f	-flight take-off -systemic AE (bronchogenic cyst)	N	-during decompression	-fatal
**Maples, 2008** [53] USA, Norfolk (Virginia)	1	32	f	-dive (60 m) -AGE	N	-during decompression	-died after several days
**Mendez, 2017** [54] Mexico, Yucatán	1	35	m	-dive (25 m for 90 min) -suspected cardiopulmonary DCS	N	-HBOT (150 min) after recompression	-fatal
**Murphy, 1991** [23] USA, Chicago (Illinois)	1	17	f	-CO-intoxication -then tension pneumothorax	N	-before and after 5 min of HBOT	-declared brain dead next day
**Quesada Suescun, 2011** [55] Spain, Santander	2	-	-	-	-	-	-fatal
**Sloan, 1989** [56] USA, Chicago (Illinois)	3	-	-	-CO-intoxication	N	-during HBOT	-
**Van Meter, 2011** [57] USA, New Orleans (Louisiana)	1	35	m	-commercial diving -hypoxia due to air supply compression	N	-before HBOT until first round of 6 ATA HBOT	-full recovery
**Weaver, 1999** [58] USA, Salt Lake City (Utah)	1	25	m	-CO-intoxication -aspiration pneumonia, cerebral edema	N	-2,9 ATA breathing O_2_	-stabilization
**Wolf, 1990** [22] USA, Durham (North Carolina)	1	69	w	-non-healing ulcer of the foot -suspected pulmonary barotrauma with air embolism	N	-defibrillation during decompression	-fatal
**Wright, 2016** [59] Micronesia, Guam (Territory of the United States)	2	27	m	-scuba diving -DCS	N	-during the third O_2_ period	-fatal
		43	m	-deep dive -DCS	N	-after recompression (due to tachypnea during fifth O_2_ period at 60 fsw)	-fatal
**Xiangdong, 2008** [60] China, Shandong	1	37	m	-poisoning due to accidental inhalation of hydrogen cyanide	N	-during HBOT	-CPR succeeded after 10 min

AAE = arterial air embolism; AE = arterial embolism; AGE = arterial gas embolism; ATA = atmospheres absolute; CA = cardiac arrest; CPR = cardiopulmonary resuscitation; CO = carbon monoxide; COT = cerebral oxygen toxicity; DCS = decompression sickness; HBOT = hyperbaric oxygen therapy; PEA = pulseless electrical activity; USA = United States of America VAE = venous air embolism; VF = ventricular fibrillation; VGE = venous gas embolism; VT = ventricular tachycardia; w/o = without; POD = postoperative day

An additional 23 papers provided information or recommendations on emergency treatment or CPR in the hyperbaric setting, which are summarized in Table 3.

Notably, certain physiological peculiarities apply in hyperbaric environments, current resuscitation guidelines will be discussed in the context of HBOT at the end of this review.

**Table 3:** Further recommendations on specific aspects of CPR during hyperbaric oxygen therapy (HBOT)

**Recommendations** **on chest compression**	♣ Immediate start of CPR [7, 14, 27, 35, 38, 45, 59, 61–63] ♣ If extraglottic device possible: 30:2, if no extraglottic device possible: compression only [42] ♣ Consider use of mechanical CPR device for evacuation [6]
**Recommendations** **on defibrillation**	♣ Precordial thump controversial [38], [37] ♣ Store defibrillator outside chamber for safety [2, 6, 7, 13, 27, 29, 35, 37, 38, 59, 61–63] ♣ Use adhesive plates [2] [28] [63] [14] ♣ 2 defibrillators certified for HBOT (Physiocontrol LifePak 1000; Corpuls3) [38]
**Recommendations** **on airway/breathing**	♣ Oropharyngeal airway [6, 27] [59, 63] with largest possible ET [61] ♣ Inflate cuffs with water or saline instead air [7] [27] [45] [61] [62] [14, 36] ♣ Video laryngoscope available inside of chamber [64] ♣ i-gel™ favorable [37, 42] ♣ Two certified HBOT ventilators (Italian Siaretron 1000 Iper [60 VF] and Maquet Servo-i HBO) [38]
**Recommendations** **on medication**	♣ i.v. lines placed before HBOT [27, 64] [59] ♣ Plastic containers have to be vented, glass bottles risk of gas embolism during decompression [38] ♣ One certified syringe pump (Pilot Hyperbaric, Fresenius Vial S.A.) [38]
**Recommendations** **on chamber type**	♣ CPR in chambers = confined spaces, potential risks increase [27, 32, 62, 65–67] ♣ Multiplace chambers with possible benefits in case of CPR during HBOT [2, 22, 45, 58, 62, 63]
**Further considerations** **and information**	♣ Interprofessional, close collaboration and training needed [2, 6, 14, 45, 64, 66] ♣ Proper examination prior to HBOT essential [6, 27, 28, 45, 68] (e.g., if Pneumothorax [7, 27, 56), drained?) as well as attention to reversible causes in case of cardiac arrest [6, 66]

CPR = cardiopulmonary resuscitation; HBOT = hyperbaric oxygen therapy; ET = endotracheal tube

## Discussion

Cardiopulmonary resuscitation during HBOT is rarely reported in the literature. Our study identified a total of 22 cases of CPR related to HBOT since 1989. The mean age was 38.8 years, which is younger than the average patient undergoing CPR in industrial countries. According to the German Resuscitation Registry (GRR) the mean age of out-of-Hospital Cardiac Arrests (OHCA) in Germany was 70.2 years in 2021 [16]. In the United States, the Cardiac Arrest Registry to Enhance Survival (CARES) registry reported a mean age of 62 years for the same observation period [17]. Although data on the number of hyperbaric treatments administered world-wide daily are unavailable, it is likely that the number of CPR events in a pressure chamber exceeds published reports.

Dieterich et al. reported 10 cases of resuscitation over a period of 17 years for their pressure chamber center in Bavaria, Germany [6]. However, CPR associated with HBOT appears to be relatively rare. Consequently, adequate preparation and education of all involved parties are crucial. Boet et al. employed a modified Delphi consensus process to identify clinical situations in hyperbaric medicine that could benefit from simulated practice. As a result, five scenarios were deemed relevant for a potential future simulated-based curriculum in hyperbaric therapy [18]:

- Seizure in the chamber.
- Fire in or immediately outside the chamber.
- Cardiac arrest in the chamber.
- Pneumothorax in the chamber.
- Technical deficiency, such as power loss while operating the chamber.

All these scenarios pose a direct or indirect life-threatening risk. Therefore, these findings underscore the importance of investigating the optimal standard for CPR in a hyperbaric chamber and should be discussed in the light of the current European Resuscitation Council (ERC) guidelines [19].

### Iatrogenic vs. non-iatrogenic cause of CPR

There are currently 14 approved applications for HBOT, such as air or gas embolism, acute thermal burn injury, or carbon monoxide poisoning [5]. In the current study, HBOT followed by CPR was primarily administered in the course of treatment of out-of-hospital events, mainly decompression sickness (DCS) and carbon monoxide intoxication. Among the DCS cases, there was only one instance of air embolism with subsequent CPR caused by hyperbaric therapy itself [20]. This is surprising, as the absolute pressure changes in hyperbaric chambers are the same as those experienced by divers. However, Wolf et al. stated that a slower rate of decompression is used after hyperbaric oxygen therapy compared to an ascending diver, which prolongs the time for gas exchange and reduces the chance of air embolism and barotrauma. Furthermore, divers are more vulnerable to developing panic, predisposing them to laryngospasm and subsequent barotrauma due to prevented exhalation [22]. Thirdly, Pendergast et al. reported an increased lung closing volume under head-out water immersion conditions, which can lead to barotrauma as a consequence of entrapped gas in the terminal airway structures [21]. Additionally, unlike most dives, hyperbaric therapy is conducted under the supervision of medical personal, who can identify potentially life-threatening symptoms and initiate immediate intervention in a well-equipped hospital environment before CPR becomes a necessity [22].

In one other case presented by Murphy et al., hyperbaric therapy led to tension pneumothorax of a young patient treated for carbon monoxide intoxication [23]. In three other cases, the underlying cause of CPR was not associated with hyperbaric therapy but still iatrogenic in nature. The majority of these cases included surgical interventions and/or catherization of arterial or venous blood vessels [6, 48, 50].

### Initiation of CPR

Early initiation of CPR is associated with a higher rate of overall survival [24, 25]. The survival rate decreases by 2.1–3.2% for every minute without CPR in the context of out-of-hospital cardiac arrest (OHCA), respectively [26]. However, immediate response to CA during HBOT may not always be feasible due to limited access to the patient and the unique environment of various pressure chambers. Timely identification of CPR need by bystanders or personal can be challenging, particularly in out-of-hospital pressure chambers like diving bells or deck-decompression chambers, which often lack real-time monitoring of patient’s vital parameters and have poor lighting conditions [29]. Due to the hyperoxic environment, pulse oximetry has limited value [27], and transcutaneous O_2_ sensors may not always be available. Monitoring of ECG and invasive blood pressure requires electrical connections to the chamber exterior [27].

Rapid assistance is crucial once CA is recognized. In spacious pressure chambers, personnel may already be beside the patient during the dive. Otherwise, the chamber must be completely decompressed before personal can access the patient, potentially taking several minutes to prevent DCS barotrauma and additional life-threatening complications. Kot et al. recommended one chamber operator and one physician per patient for monoplace chambers and one chamber operator, up to two internal attendees and one physician per patient for multiplace chambers [28]. However, small monoplace chambers, diving bells, and deck-decompression chambers present challenges due to confined space. Acott et al. described the time-consuming procedure of recovering an unconscious diver into a diving bell, which involves flooding the bell, gearing up, retrieving the diver, and evacuating the bell. Once inside the bell with its hatch closed, access to the diver for CPR initiation remains difficult, as the diver usually lies in a upright position against the bell wall with limited access for resuscitation in every spatial dimension [29].

#### Recommendation

The interior of the chamber should be equipped with adequate lighting conditions. Monitoring of vital parameters should minimally include blood oxygen saturation using transcutaneous O_2_ sensors and ECG, and be expanded as necessary for patients requiring intensive care treatment. Depending on the type of pressure chamber, a specific action plan should be developed, detailing concrete steps for initiating CPR. This may include emergency decompression of the chamber, recruiting sufficient personnel to recompress and enter the chamber for CPR (see Table 3). Regular training for HBOT practitioners should incorporate these adapted guidelines to ensure preparedness in the event of CA during HBOT.

### Chest compression

In addition to the timely initiation of CPR, the quality of CPR is a crucial factor for overall survival. The American Heart Association (AHA) quality criteria for CPR include [30, 31]:

- Minimize interruption.
- Aim for chest compression fraction above 80%.
- Chest compression rate 100–120/min.
- Compression depth at least 5 cm in adults.
- Full chest recoil.
- Avoid excessive ventilation.

The majority of case reports identified in this review did not provide detailed information on CPR parameters or the adopted CPR algorithm. Nevertheless, CPR may have been performed according to the AHA or ERC (European Resuscitation Council) guidelines. Acott et al. noted that the resuscitator´s position depends on the chamber size and shape, discussing an upright position CPR in a diving bell due to confined space and the patient hanging from a pulley attached to the bell´s interior wall. They argued that efficient chest compressions (CC) in supine position can typically achieve up to 30% of cardiac output, and therefore, CPR with the patient in an upright hanging position is considered inefficient concerning cerebral perfusion and should not be attempted [29]. Bhatnagar et al. identified two CC techniques suitable for confined spaces: one with the rescuer positioned over the patient’s head and one with the rescuer sitting on top of the patient with both legs straddled [32]. If the CPR technique does not allow easy access to the patient’s airways, the 30:2 algorithm should be abandoned, and a compression-only CPR performed [29]. Given the hyperbaric hyperoxygenation typically administered to patients inside the chamber, the benefits of intermediate ventilation compared to chest compression only may be significantly reduced, at least during the first five minutes after cardiac arrest. In this context, maintaining sufficient circulation to transport oxygen to organs should be of upmost importance [6]. However, this contrasts with a study by Weaver et al., who reported two cases of hypoxemia during the first air-breathing period of the applied HBOT scheme [33]. The authors emphasized that patients with severe lung dysfunction and a high degree of venous admixture can become hypoxic despite hyperbaric therapy [33]. Furthermore, Dieterich et al. recommended the use of a mechanical resuscitation aid if a patient still requires resuscitation after decompression to avoid significant delays in CPR during transport out of the chamber [6].

#### Recommendation

CPR should be performed in a supine position on a solid surface. Several CC techniques, such as the straddle variant, can be executed in confined spaces. If 100% oxygen was administered, it might be beneficial to forgo intermittent ventilation in favour of continuous CC. Regular training for HBOT practitioners should integrate these adaptations to ensure preparedness and improve patient outcomes in the event of cardiac arrest during HBOT.

### Defibrillation

Ventricular fibrillation (VF) is the main cause of cardiac arrest, for which the most effective treatment is defibrillation [34]. In the context of OHCA, the survival rate decreases by 2.1% for every minute without defibrillation [26]. However, during HBOT, administering high voltage pulses in a hyperoxygenated environment presents significant safety hazards [35]. Several studies argue that the use of portable defibrillators inside the chamber poses risks, such as [6, 35]:

- Potential fire due to high voltage electronics.
- Implosion of the vacuum-filled CRT monitors.
- Device malfunction under hyperbaric conditions.
- Operator error resulting from nitrogen narcosis or decompression sickness.

In general, placing the defibrillator outside the chamber is recommended. The main device is connected to the chest paddles inside the chamber via a penetrator through the chamber wall, allowing both heart rhythm monitoring and electric shock application. Dieterich et al. demonstrated in their simulation training that even without pre-attached patches, the time between CA recognition and the first shock was within a reasonable two-minute period for unanticipated defibrillation cases [6]. Monoplace chambers should be switched from oxygen to ambient air during decompression. If this is not possible, at least 40 s must elapse after opening the chamber for the oxygen to dissipate before defibrillation. Oxygen-enriched garments and other textiles should be removed before shock application, even after decompression [36]. The risk of current transmission to bystanders during shock application is significantly increased. Ensuring proper sufficient earthing of the chamber and the use of grounding footwear can further contribute to staff safety. Biphasic defibrillators, which deliver the same (or better) therapeutic effect compared to monophasic devices using less electrical energy, should be preferred during hyperbaric therapy [35].

Acott et al. discussed performing a precordial thumb for confined spaces with difficulties in providing BLS (e.g., DB or DDC). According to the ERC, this technique is recommended only in witnessed and monitored VF or VT within the first 15 s of recognition and only in the absence of a defibrillator. The AHA neither explicitly supports nor opposes the use of the precordial thump. Some authors do not recommend its use [37], while others consider a precordial thumb in case of witnessed VT or VF in absence of a defibrillator [6].

#### Recommendation

The risk of fire due to defibrillation is of utmost concern during HBOT. Defibrillator should not be stored inside the pressure chamber. The use of biphasic devices placed outside the chamber with connection to chest panels inside should be preferred. If defibrillation is necessary, an FiO_2_ of 21.5% should not be exceeded at the moment of shock application [38]. Adequate earthing of the chamber, grounding footwear, and maintaining a sufficient distance from the patient must be ensured to avoid current transmission.

### Airway management

The optimal airway technique for both out-of-hospital and in-hospital resuscitation remains uncertain [39]. Current guidelines recommend an airway management approach based on patient factors, personnel skills, and the stage of resuscitation [40]. In the context of hyperbaric therapy, additional factors such as number of staff, hyperoxygenation, and confined space must be considered. The Diving Medical Advisory Council (DMAC) publishes a list of medical equipment to be held in a diving bell, which includes the following airway equipment [41]:

- Resuscitation face masks.
- 2 Guedel tubes sizes 3 and 4.
- 1 or 2 supraglottic airway devices.

Acott at al. tested the i-gel® laryngeal mask under hyperbaric conditions and found no changes in consistency or development of bubbles within its thermoplastic material during pressure chamber dives with an atmospheric pressure up to 608 kPa. Unlike other airway devices, the i-gel® does not require an inflatable cuff, eliminating the risk of tissue compression or leakage due to changes in ambient pressure. Given its ease of administration even for untrained or non-medical personal, the authors recommended the i-gel® for usage in resuscitation [42]. Dieterich et al. emphasized the importance of securing the airway within 5 min after initiation of resuscitation for in-hospital pressure chambers [6]. However, they highlighted the importance of continuous cuff pressure monitoring to prevent oxygen leakage (which is particularly relevant in the context of defibrillation). Supraglottic airway devices should be used if endotracheal intubation fails after the first attempt. If resuscitation begins during an oxygen “break” or during decompression, the time until the airways is secured should not exceed three to four minutes.

#### Recommendation

If feasible, the airway should be secured with an endotracheal tube within 5 min after CA was detected. If endotracheal intubation would disproportionately delay the process, a laryngeal mask offers an alternative that can be administered quickly and with easily handling in terms of ambient pressure changes. The i-gel® laryngeal mask, in particular, has demonstrated utility and stability under hyperbaric conditions, making it a suitable option for airway management during hyperbaric therapy. According to evidence from difficult airway studies [43], it should be recommended to also ensure availability of video laryngoscopy (VL) in case of CPR during HBOT.

### Medication

Unlike chest compressions or defibrillation, the efficacy of drugs during resuscitation is still considered contradictory [44]. As of HBOT, the current study found no peculiarities for drug use in pressure chambers. However, there are pitfalls on how to properly store and administer medication, especially in an emergency situation. The confined environment within many pressure chambers requires a standardized storage concept, which should be taught to all medical personal [6]. Lind referred to the fact that continuous infusions (especially inotrope and vasopressor drugs) should be administered via reliable battery-power infusion pump systems that are approved for HBOT. Changes in ambient pressure should be accompanied by increased attention to the infusion pumps, as remaining gas in the syringe and/or tubing can cease drug delivery undetected by the syringe pump. As for monoplace chambers, infusion pumps are primarily located outside the chamber. Thus, drug delivery can become inaccurate since the pump must overcome the chamber overpressure [45]. Other authors highlighted the fact that both plastic and glass infusion bottles should be vented to prevent the risk of gas embolism and explosion (e.g., during rapid decompression) [27].

#### Recommendation

Establishing a proper IV line should be done prior to HBOT. In case of drug administration during HBOT with no IV line or impossibility to establish, an IO line should be applied. Drug therapy should be administered according to the ERC or AHA guidelines. Plastic and infusion bottles must be properly vented during usage to avoid gas embolisms. Glass bottles are not recommended due to risk of air embolism. Attention should be given to infusion pumps during changes in ambient pressure to ensure accurate drug delivery. Staff should be trained in the standardized storage and administration of medications in the confined environment of pressure chambers to ensure proper handling during emergency situations.

### Further considerations

**Fig. 2:**
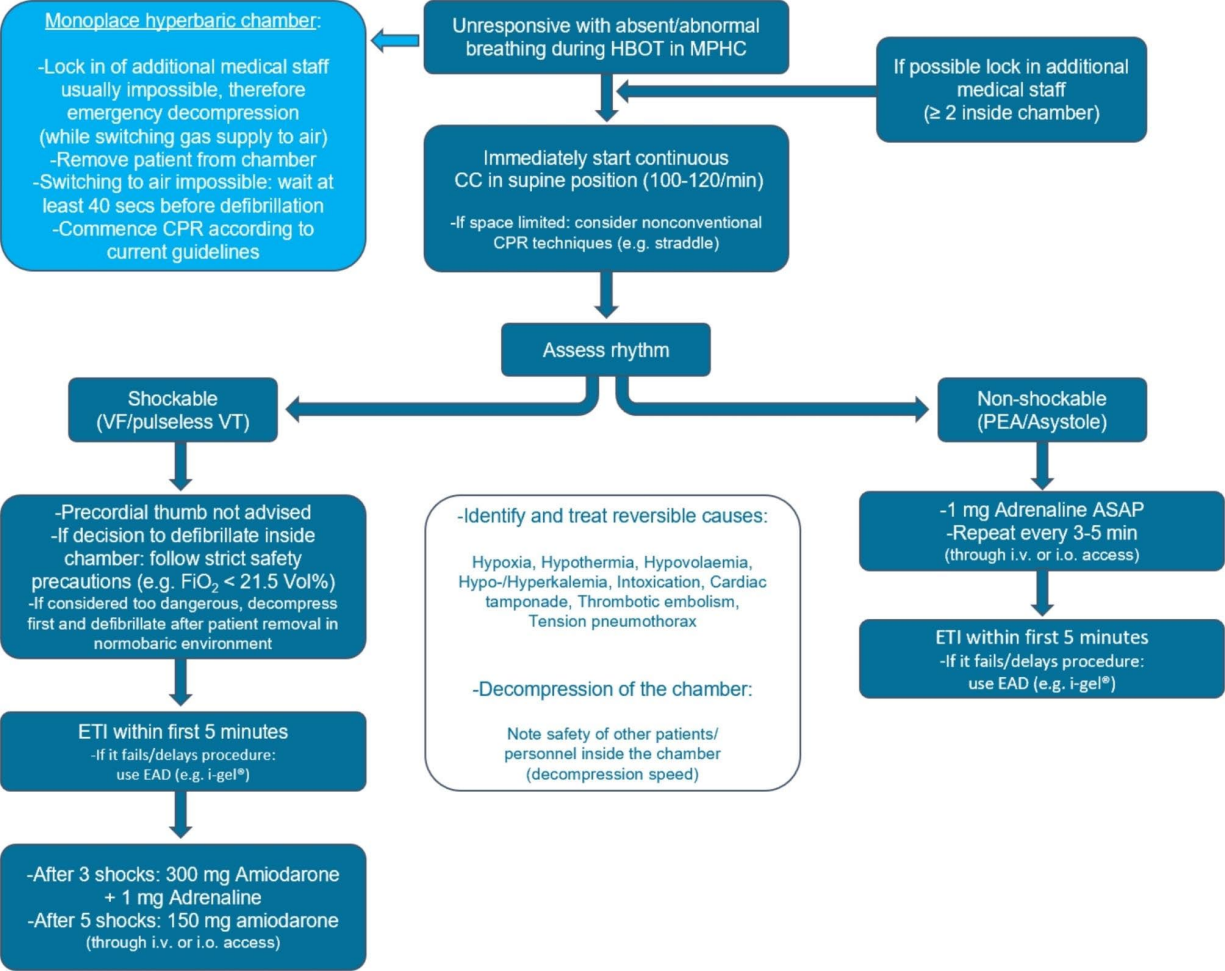
**– Modified ERC Advanced Life Support algorithm for CPR in the hyperbaric setting** Algorithm modified according to the European Resuscitation Council (ERC) Advanced Life Support (ALS) algorithm and the resuscitation algorithm modified for hyperbaric conditions from Dieterich et al. [6] ASAP = as soon as possible; CC = chest compression; CPR = cardiopulmonary resuscitation; EAD = extraglottic airway device; ETI = endotracheal intubation; FiO2 = fraction of inspired oxygen; HBOT = hyperbaric oxygen therapy; i.v. = intravenous access; i.o. = intraosseous access; MPHC = multiplace hyperbaric chamber; PEA = pulseless electrical activity; VF = ventricular fibrillation; VT = ventricular tachycardia

The presence of medical personnel inside a pressure chamber inherently carries the risk of incapacitation of the staff themselves. Special attention should be given to potential dangers such as DCS or barotrauma, especially during emergency decompression while performing physically demanding CC [45]. Effective communication between personnel outside and inside the chamber is crucial during emergency situations and should be practiced regularly through simulation trainings. For further considerations concerning CA during HBOT see also Fig. 2. Post ROSC therapy should follow ERC/AHA recommendations as there is currently no evidence about a specific post ROSC treatment after CPR during HBOT.

## Conclusion

The exact number of hyperbaric treatments conducted worldwide each year is currently unknown. Nevertheless, resuscitation associated with HBOT appears to be fairly rare, yet remains a life-threatening event. The pressurized chamber environment presents unique challenges to every aspect of the CPR algorithm. The confined space imposes limitation on movement and storage, while dynamic pressure conditions pose potential hazards to personnel, patient, and medical equipment. Fluctuating oxygen partial pressure necessitate continuous monitoring of the patient’s oxygen status and is associated with an increased risk of fire. Moreover, both patients and staff are isolated from the outside world within the pressure chamber, making effective verbal communication particularly crucial.

These factors lead to the essential requirement that all personnel involved in HBOT must thoroughly learn CPR procedures theoretically and practice them hands-on in advance. This work contributes significantly to supplementing the “CPR under special circumstances” chapter, offering valuable insights and recommendations for healthcare professionals working in hyperbaric environments.

## Appendix

Search string:

*(pressure chamber OR decompression OR hyperbaric) AND (cardiac arrest OR cardiopulmonary resuscitation OR advanced life support OR ALS OR life support OR chest compression OR ventricular fibrillation OR heart arrest OR heart massage OR resuscitation)*.

Search string adjusted by EMBASE:

*(‘pressure chamber’/exp OR ‘pressure chamber’ OR ((‘pressure’/exp OR pressure) AND (‘chamber’/exp OR chamber)) OR ‘decompression’/exp OR decompression OR hyperbaric) AND (‘cardiac arrest’/exp OR ‘cardiac arrest’ OR ((‘cardiac’/exp OR cardiac) AND (‘arrest’/exp OR arrest)) OR ‘cardiopulmonary resuscitation’/exp OR ‘cardiopulmonary resuscitation’ OR (cardiopulmonary AND (‘resuscitation’/exp OR resuscitation)) OR ‘advanced life support’/exp OR ‘advanced life support’ OR (advanced AND (‘life’/exp OR life) AND (‘support’/exp OR support)) OR als OR ‘life support’ OR ((‘life’/exp OR life) AND (‘support’/exp OR support)) OR ‘chest compression’/exp OR ‘chest compression’ OR ((‘chest’/exp OR chest) AND (‘compression’/exp OR compression)) OR ‘ventricular fibrillation’/exp OR ‘ventricular fibrillation’ OR (ventricular AND (‘fibrillation’/exp OR fibrillation)) OR ‘heart arrest’/exp OR ‘heart arrest’ OR ((‘heart’/exp OR heart) AND (‘arrest’/exp OR arrest)) OR ‘heart massage’/exp OR ‘heart massage’ OR ((‘heart’/exp OR heart) AND (‘massage’/exp OR massage)) OR ‘resuscitation’/exp OR resuscitation)*.
